# Laboratory investigation and core flood demonstration of enhanced biogenic methane generation from lignite

**DOI:** 10.3389/fbioe.2024.1308308

**Published:** 2024-02-19

**Authors:** Priyanka Basera, Meeta Lavania, Nimmi Singh, Banwari Lal

**Affiliations:** ^1^ The Energy and Resources Institute (TERI), New Delhi, India; ^2^ Ongc Energy Centre, Delhi, India

**Keywords:** lignite, volatile fatty acids, methane, anaerobic scale up, coreflood experiment

## Abstract

Over the last several decades, coalbed methane (CBM) has emerged as an important energy source in developing nations like India as well as worldwide and is expected to play a significant role in the energy portfolio of the future. The current scenario of rapid exhaustion of fossil fuels is leading to the need to explore alternative and efficient fuel resources. The present study demonstrates enhanced methane production per gram of lignite (lowest-rank coal). Optimization of the bioconversion of lignite to methane revealed 55°C temperature and 1.5 g/L NaCl concentration as ambient conditions for the process. A scale-up study in the optimized condition showed 2,800 mM methane production per 25 g of lignite in anaerobic conditions. Further, Fourier transform Infrared (FTIR) and Gas Chromatography Mass Spectrometry (GCMS) analysis showed bioconversion of lignite into simpler intermediate substrates required for methane production. The results highlighted that the bacterial action first converts lignite into volatile fatty acids, which subsequently get converted into methane. Further, the exploration of indigenous microbial consortia in Tharad well (THAA) mainly comprises the order Methanosarcinales and Methanomicrobiales. The pathogenicity of the microbial consortium THAA was declared safe for use in mice via the oral route by The Energy and Resources Institute (TERI), India. The study demonstrated the development of indigenous consortia (TERI THAA), which can potentially enhance methane production from the lowest coal grade under extreme conditions in Indian coal beds.

## Graphical Abstract

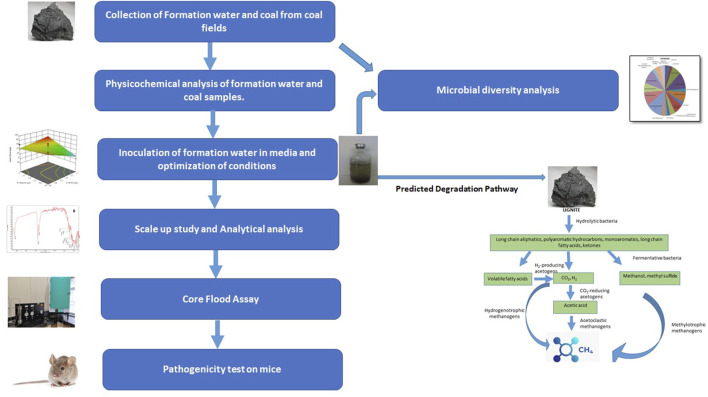

## 1 Introduction

Petroleum-based hydrocarbons are a major energy source used worldwide (Litvinenko, 2020). Coal is the world’s largest and most prevalent conventional energy, mainly categorized into four main types or ranks: anthracite, bituminous, sub-bituminous, and lignite. The lignite and sub-bituminous coals are the low-ranked coal with low commercial and calorific values (Flores, 2014; Schobert, 2017). The combustion of coal leads to the release of toxic and harmful substances, which negatively impacts the human habitat and environment (Goodwin, 2014; Perera, 2017). Therefore, with a constantly increasing demand and finite fossil fuel sources, there is a surging demand to seek an alternate source of hydrocarbons.

The abundance of lignite and sub-bituminous coal accounts for 53% of the total coal resources in the world. According to the report of Ministry of Coal, the all India Production of coal during 2022-23 was 893.19 MT with a positive growth of 14.77% (https://coal.gov.in/en/major-statistics/production-and-supplies). According to the Geological Survey of India, the total geological resource of lignite of the country stands at 45,758.70 million tonne as on 1 April 2019 and major deposits are located in the state of Tamil Nadu, followed by Rajasthan, Gujarat, Kerala, West Bengal, Union Territory of Jammu & Kashmir and, Pondicherry (Fan et al., 2019). Though India has significant lignite reserves, the majority of these are located in environmentally sensitive geographical areas or at a depth of economically unviable options for exploration. Moreover, the high moisture content, low calorific value, and spontaneous combustion of lignite are the factors responsible for its lower economical utilization (Fan et al., 2019). Therefore, it is important to study how to utilize lignite resources (conventional and unconventional) present in India and worldwide to bridge the gap between demand and supply.

Production of biogenic coalbed gas has been reported from coal reserves in many countries (Goraya et al., 2019). This motivates the effective utilization and understanding of environmentally friendly method: biological methane production from coal. Previous studies have shown that “methane producing” consortium extracted from coal was observed to grow on low-volatile bituminous coal as a sole carbon source (Thielemann et al., 2004). A microbial consortium was successfully applied to bioassay methane generating potential in 18 coal samples (Jones et al., 2008).

Biogenic methane production is the consequence of microbial action leading to complex biochemical reactions taking place during the decomposition of organic matter in anoxic environments. It has been noted that all coal types contain gases, with methane typically being the major constituent (90%–97% of all gases), regardless of maturity, organic makeup, or nature of occurrence. Most appropriate for commercial methane extraction are deep-seated seams (depth of roughly 1,000 m or more) having coal of higher rank (carbon content >83.5%, Vitrinite reflectance R_0_ max 0.7% and above). There are several pathways using which the methanogens produce methane (Boone, 2000). The most prevalent mode of methane production is a hydrogenotrophic pathway which is the hydrogen-mediated reduction of carbon dioxide. Various environmental factors like pH, salinity, temperature and nutritional factors (inorganic and organic) can significantly affect methane production from microbes (Boone, 2000). For instance, methane synthesis is limited in conditions with slow rates of organic matter turnover because the hydrolysis of big polymers typically limits microbial activity in anoxic environments, and low molecular weight intermediates do not accumulate significantly (Menzel et al., 2020; Mulat et al., 2018). Despite being extremely complex and poorly understood, biogenic methane generation pathways are widespread in nature (Basera et al., 2022). It is believed that 20% of the natural gas in the earth is from methanogens, of which two-thirds are from acetate fermentation and one-third from carbon dioxide reduction (Enzmann et al., 2018; Schöne and Rother, 2018). Several studies have reported coal degradation by microbial cultures to produce methane (Iram et al., 2017). Preliminary evidence suggests that lignite can be microbially degraded and biologically converted to methane (biological pathway). Chemical and isotopic data have established that anaerobic lignite breakdown results in the formation of biogenic methane (Yoon et al., 2016). Anaerobic lignite degradation, leading to biogenic methane generation, has been demonstrated using chemical and isotopic evidence (Yoon et al., 2016). Because it is a rich source of complex organic materials, lignite serves as a substrate for microbial decomposition. However, it is a challenging microbiological substrate due to complex aromatic compounds and macromolecules produced from lignin in the coal matrix. The complex process of degrading lignite to methane by microbes involves the initial disintegration of the lignite’s macromolecular matrix and the fermentation of huge intermediates to simpler organic molecules, allowing them to undergo additional transition to produce methane (Wang et al., 2017).

However, all the previous studies on biogenic methane production from lignite conducted in the laboratory were under mesophilic conditions. But, generally, many unminable lignite reserves in India are high in temperature (>50°C). Such thermophilic lignite reserves are a potent candidate for biogenic methane production for Coal bed methane (CBM). Therefore there is a need to direct the research toward biogenic methane production from sub-surface lignite at such high-temperature conditions (55°C–60°C). With the increasing fuel prices and economic forecasting, this technology is expected to significantly contribute towards producing alternative gaseous fuel.

The present study provides important insights into the bioconversion of lignite to methane by exploring the microbial diversity of the consortia involved in methanogenesis. Further optimization of the conditions was also conducted for enhanced recovery of gases. The findings of the study will contribute significantly to paving future directions in the related field.

## 2 Materials and methods

### 2.1 Sample collection and onsite inoculation

During the drilling of wells of Mehsana, Ahmedabad, and Patan-Tharad area of Gujarat, fresh lignite core and water samples were collected from the different lignite seams. The first phase of sampling (December 2017) included the collection of formation water samples from different depths of the well located in Tharad (THAA). In the second phase of sampling (January 2018), lignite and formation water samples were collected from different depths of the well in Langnaj (LJAY).

All the samples were inoculated with formation water onsite in specific anaerobic methanogenic media (MPB) broth followed by storing at 4°C and then transported to the laboratory for further analysis. The composition of MPB culture medium (g/l in deionized water) used: K_2_HPO_4_—0.3; KH_2_PO_4_—0.3; NH_4_Cl—0.5; MgSO_4_.6H_2_O—0.2; NaCl—1.0; yeast extract—1.0; casein peptone—1.0; resazurin—0.001; L-cysteine HCl—0.5 (pH 7.00 ± 0.2) (Basera et al., 2022).

### 2.2 Physico-chemical analysis of coal and formation water

The proximate and ultimate analysis of coal from wells (THAA and LJAY), in the context of ash, moisture, volatile matter, and fixed carbon, along with the specific Carbon, Hydrogen, Nitrogen, Sulphur, and Oxygen (CHNSO) profile, were determined as per the guideline of Bureau of Indian Standards. The method followed and the reference number is mentioned in Test method column of Table 1. The physicochemical analysis of the formation water samples was done. The presence of heavy metals such as Arsenic, Cadmium, Chromium, Copper, Zinc, Copper, and Mercury was also estimated as per the Bureau of Indian Standards and American Public health association standard methods (Rajkovic et al., 2008). The reference of the method followed is described in Table 2.

**TABLE 1:** Ultimate and proximate analysis of collected Lignite samples.

Test	Method	THAA	LJAY
Ash (%)	IS:1350 (P-1)1984	3.92	6.63
Moisture (%)	IS:1350 (P-1)1984	19.16	20.33
Volatile Matter (%)	IS:1350 (P-1)1984	46.77	31.21
Fixed Carbon (%)	IS:1350 (P-1)1984	30.15	41.83
			
Carbon (%)	IS:1350 (P-4/sec-1)1974	58.76	53.95
Hydrogen (%)	IS:1350 (P-4/sec-1)1974	4.98	3.97
Nitrogen (%)	IS:1350 (P-4/sec-1)1974	0.57	0.01
Sulfur (%)	IS:1350 (P-3)1969	0.48	0.55
Oxygen (%)	IS:1350 (P-4/sec 1)1974	12.13	14.56

**TABLE 2:** Physico-chemical analysis of formation water from THAA (Tharad), and LJAY (Langnaj) wells.

Test Parameter	Test Methods	Sample THAA well	Sample LJAY well
pH		6.86	6.86
Salinity (g/l)		22.23	22.23
COD (mg/l)	IS: 3025(PT-58):2006	163.26	1926.08
BOD (mg/l)	IS: 3025 (PT-44):1993	60	700
Total suspended solid (mg/l)	IS: 3025 (PT-15):1984	78	44
Total organic carbon, (TOC, mg/l)	APHA-5310	19.4	19
Dissolved organic carbon (DOC, mg/l)	APHA5310	12.8	16
Total dissolved solid (mg/l)	IS: 3025 (PT-16):1984	15788	182.48
Chloride (mg/l)	IS: 3025 (PT-32):1988 (Cl-2)	9659.41	3208.93
Sulphate (mg/l)	IS: 3025 (PT-24):1986(Cl-4)	18.21	175.89
Fluoride (mg/l)	APHA-4500-F-D	5.59	0.95
Copper (mg/l)	IS: 3025 (PT-2):2004	<0.01	0.72
Arsenic (mg/l)	IS: 3025 (PT-37):1988 (Cl-2)	<0.01	<0.01
Zinc (mg/l)	IS: 3025 (PT-2):2004	<0.01	1.2
Total Iron (mg/l)	IS: 3025 (PT-53):2003(Cl-6)	0.2	2.7
Total Chromium (mg/l)	IS: 3025 (PT-2):2004	<0.01	<0.01
Cadmium (mg/l)	IS: 3025 (PT-2):2004	<0.01	<0.01
Silver (mg/l)	IS: 3025 (PT-2):2004	<0.01	<0.01
Nickel (mg/l)	IS: 3025 (PT-2):2004	<0.01	0.09
Carbon (%)	By CH analyzer	0.05	0.032
Hydrogen (%)	By CH analyzer	0.02	0.012
Nitrogen (%)	IS:3025 (Pt-34)1988	0.002	0.01
Sulfur (%)	IS:3025 (Pt-24)1986 (Cl.4)	<0.001	<0.001

### 2.3 Enrichment of indigenous methanogenic consortia

To separate and characterise methanogens from the formation water samples, an enrichment approach was used. A volume of 45 mL of medium was dispensed into each 130 mL serum bottle flushed with O_2_-free N_2_. The serum bottles were sealed with butyl rubber stoppers and crimped with aluminum seals. Before inoculation, the sealed, pressurized bottles were sterilized in an autoclave at 121°C temperature for 15 min. At time zero, 45 mL of culture medium was inoculated with 5 mL of the formation fluid/culture using aseptic, strict anaerobic techniques and incubated at 55°C (reservoir’s bottom hole temperature) for 20 days. After the first enrichment cycle was completed, the enriched cultures were transferred to a fresh media (MPB) containing lignite (1% w/v) as a carbon source and incubated for 21 days.

Based on the initial analysis (concerning methane production) of the consortia obtained from formation water, the microbial consortia recovered from the Tharad well were selected for further studies (Supplementary Figure S1). The consortium was also tested for its efficiency in converting coal into methane at different temperatures and salinity (NaCl) conditions.

### 2.4 Analysis of microbial community in formation water and identification of consortia

With the aim of identifying the microbial community in the formation water, the total genomic DNA was extracted from the formation water, 500 mL of water sample was passed through a membrane filter (90 mm, 0.22 μm). The formation water contained suspended coal particles. To avoid losing those microorganisms attached to coal particles, all that was retained on the filter were then processed for DNA extraction using Power water DNA extraction kit (Mo Bio, United States). DNA was extracted as per the manufacturer’s protocol. Following extraction, DNA samples were quantified and evaluated using Nanodrop. The extracted DNA sample with good quality (A260/A280: 1.8–2.0) and concentrations (more than 50 ng/μL) was taken up further for sequencing.

Polymerase chain reaction (PCR) amplification was done using primers for 16S rRNA (338F: ACT​CCT​ACG​GGA​GGC​AGC​A, 806R: GGACTACHVGGGTWTCTAAT, targeting the V3-V4 region) (Caporaso et al., 2010). Amplicon libraries were prepared with high-quality DNA and Nextera Index Kit 16S metagenomic sequencing library preparation protocol. Libraries were sequenced using the Illumina MiSeq platform with 2*300 base-pair chemistry at Medgenome Pvt. Ltd., Bangalore, India.

FASTQC tool v 0.11.8 (Babraham bioinformatics) verified the quality of sequences after demultiplexing and adaptor/primer/barcodes sequence removal from raw reads. Merging of paired-end reads of each sample was carried out using FLASH v 1.2.11 software (Magoč and Salzberg, 2011). Quantitative Insights Into Microbial Ecology (QIIME 2) standard protocol (Bolyen et al., 2019) was followed for quality filtering, library preparation, and Operational Taxonomic Units (OTU) picking. DNA sequences with more than 97% similarity were assembled into the same operational taxonomic units (OTUs). OTU selection and taxonomic assignment were performed according to the SILVA reference database version 138.

### 2.5 Optimization of lab conditions for enhanced methane production

Physico-chemical parameters greatly impact biogenic methane production (Goswami et al., 2016). Therefore the optimization of laboratory conditions is necessary to study their impact on microbial activity.

The influence of temperature and salt concentrations were analysed by measuring methane production at different conditions. The analysis of the effect of variable concentrations of NaCl was done at different concentrations of 0.5, 1.0, and 1.5 g/L and was checked at selected temperatures of 55°C, 60°C, and 70°C. The biogenic production of methane was analyzed at each condition. The concentration and temperature at which the methane production was found to be highest were chosen for further experiments.

### 2.6 Analytical methods

#### 2.6.1 Gas analysis and VFA analysis

Production of the gases in the headspace (hydrogen, methane, carbon dioxide, and nitrogen) was quantified by gas chromatography GC 7890A (Agilent Ltd. United States), equipped with a molecular sieve packed stainless steel column (2 m × 2 mm id NUCON, INDIA) and a thermal conductivity detector (TCD) (Rathi et al., 2015). Argon was used as the carrier gas at a flow rate of 1.0 mL/min, and the operating temperatures of the injector, oven, and detector were taken to be at 100, 50, and 150°C, respectively (Rathi et al., 2015). During the enrichment process, methane production in incubated cultures bottle were tested with regular interval of 10 days in each enrichment cycle by taking 0.5 mL of headspace gas samples from the anaerobic serum bottles using gas-tight syringe. The expected headspace gases such as methane, hydrogen, carbon dioxide and nitrogen were quantified by gas chromatography and method was calibrated by injecting standard gases (Sigma) with the range of concentration (0.1 mL, 0.2 mL, 0.3 mL, 0.4 mL, and 0.5 mL). The calibration curve was obtained for all the standards with R2 approximately 0.998. The measured LOD is 0.08 ppm and LOQ is 0.170 ppm.

The water samples were also analyzed for volatile fatty acids (VFAs) and CHNS analysis, The concentration and composition of VFA (formic, acetic, butyric, propionic, valeric, isovaleric, hexanoic and heptanoic acids) in the effluents were analyzed by gas chromatography (GC 6890, Agilent) with a flame ionization detector. The column (CP-Sil 5 CB, 25 m × 0.32 mm × 5 μm, Agilent) operating temperature profiles for 70°C for 0.5 min, then increased to 200°C at 5°C/min and then to 250°C at 10°C/min hold for 1 min. The injector and detector temperature were 250°C. Volatile Free Acid Mix (SupelCo, United States) was used as a VFA standard.

#### 2.6.2 Detection of hydrocarbon

Hydrocarbon were measured in GC 6890 (Agilent Technologies) equipped with HP-5 (5% phenylmethyl siloxane) capillary column (30 m × 320 μm × 0.5 µm) and helium (5 mL min-1) as the carrier gas was used for the analysis of aliphatic and aromatic fractions. The injector and detector (flame ionization detector) temperatures were 280°C and 300°C respectively. The oven was programmed to rise from 80°C to 300°C with an increment of 5°C min-1 and then held for 30 min at this temperature. Authentic standards for aliphatic (C14-C38) and aromatic (naphthalene, anthracene, fluoranthene, pyrene, benzo [a] anthracene, chrysene, benzo [b] fluoranthene, benzo [k] fluoranthene and dibenzo [b] anthracene) were used as reference (Sigma).

#### 2.6.3 Fourier transform infrared spectroscopy (FTIR)

FTIR was carried out to identify the functional groups present in the microbially degraded coal sample. The functional group was characterized by using Fourier Transform Infrared Spectroscopy (Perkin Elmer). All spectra were recorded in an absorbance scale with a mid-measuring region of 400–4,000 cm^−1^ (mid-infrared range). The resolution was set at 4 cm^−1^ with 64 scans per spectrum (Sharma et al., 2020).

#### 2.6.4 Scanning electron microscopy (SEM)

The interaction between microbial species and coal was visualized with the help of Scanning Electron Microscopy (Carl Zeiss, Germany). Under aseptic conditions, the sample was absorbed for 2–4 h in a 2.5% glutaraldehyde solution. Phosphate buffer (0.1 M) was used for primary washing, with a pH of 7.2. The sample was dehydrated using ethanol solution in a series of 10%–100% followed by acetone. The overnight air-dried sample was then coated with gold-palladium and visualized through SEM (10 KV in Zeiss EVO MA 10).

### 2.7 Optimization of the nutrient recipe for enhanced biogenic methane generation from lignite

Response surface methodology (RSM) using Box Behnken design was applied to optimize the three important medium components, namely ammonium chloride (0.5–2 g/L; A), casein peptone (0.5–2 g/L; B), and yeast extract (0.5–2 g/L; C) of MPB medium. All experiments were performed in 130 mL serum bottles with a working volume of 45 mL of liquid anaerobic medium with 0.5 g of ground coal and 5 mL of inoculum (enriched media), incubated at 55°C. After 20 days of incubation, methane production was studied as described. All the experiments were performed in triplicate. The data points are the average of the triplicate ±standard deviation (less than 5% of the average), and the calculated significance *p*-values are ≤0.01.

Utilizing Response Surface Methodology (RSM), optimization studies for increased methane generation from coal were created experimentally (Arslan-Alaton et al., 2010). RSM was used to assess the association between independent variables and biologically improved methane generation (Gopal et al., 2021). The primary composite design model is the one employed in numerical experiments. The model used for numerical experiments is a central composite design. Three major essential nutrients (Yeast Extract, Peptone, and ammonium chloride) were taken in Table 3.

**TABLE 3:** Description of factors and their variable ranges.

Factor	Name	Variable range	−1	0	+1	+2
A.	Yeast Extract	0.5–2 gm/L	0.5	1	1.5	2
B.	Peptone	0.5–2 gm/L	0.5	1	1.5	2
C.	Ammonium chloride	0.5–2 gm/L	0.5	1	1.5	2

Table 3 describes the range of variable concentrations designed for the optimization of methane production. For the above-mentioned four factors, a total of 30 experiments (Table 4) were designed based on response surface models (Central Composite Design). Two variables, such as yeast extract and concentration of ammonium chloride, have a distinct effect on the production of biological methane and volatile fatty acid production from lignite. For all factors, the interactive effect was identified to be insignificant (*p* > 0.05). In a polynomial equation, the correlation coefficient (R^2^) was calculated as 0.9940 for the production of methane, which indicates the variability of the observed response values.

**TABLE 4:** 30 runs central composite RSM Design table.

Run order	Yeast Extract (g/L)	Ammonium chloride (g/L)	Peptone (g/L)
1.	0.5	0.5	2
2.	2	0.5	2
3.	2	0.5	2
4.	1.25	1.25	1.25
5.	0.5	0.5	0.5
6.	2	0.5	0.5
7.	1.25	1.25	1.25
8.	1.25	2	1.25
9.	1.25	1.25	1.25
10.	0.5	2	0.5
11.	1.25	1.25	1.25
12.	0.5	0.5	0.5
13.	1.25	0.5	1.25
14.	2	2	0.5
15.	2	1.25	1.25
16.	0.5	0.5	2
17.	2	0.5	0.5
18.	0.5	1.25	1.25
19.	1.25	1.25	1.25
20.	0.5	2	0.5
21.	2	2	2
22.	1.25	1.25	2
23.	1.25	1.25	1.25
24.	1.25	1.25	1.25
25.	1.25	1.25	1.25
26.	0.5	2	2
27.	0.5	2	2
28.	1.25	1.25	0.5
29.	2	2	2
30.	2	2	0.5

### 2.8 Scale-up experiment

Scale-up studies were conducted to study the efficacy of Tharad consortia for methane production. The start-up culture was prepared with 1,000 mL of the liquid MPB medium and lignite (1% w/v) as substrate. The sterile nutrient medium was inoculated with 5 mL of the Tharad culture under aseptic conditions and incubated at 55°C for 20 days, after which it was checked for methane production (as described in Section 2.6). After 20th day, 150 mL grown culture was transferred in a 5 L anaerobic bottle containing 2.5 L of liquid MPB medium containing lignite (1% w/v) as the carbon source under strictly anaerobic conditions. The inoculated bottle was then incubated at 55°C for 20 days, post which the methane production was quantified.

### 2.9 Core flood assay

The core-flood experiment was set up to examine the feasibility of lignite bioconversion to methane using THAA consortia when exposed to temperature and pressure conditions. The study was conducted using the core-flood apparatus (Figure 6A) at a pressure of 900–940 psi and 55°C temperature. Before setting-up up the core-flood study, the internal tubing of the apparatus was flushed with nitrogen gas (99.9% purity) to get rid of atmospheric oxygen. A total of 0.65 gm of lignite core/block was powdered and placed inside the core holder. The core holder was then flooded with ∼650 mL of the medium containing the inoculum. Nitrogen gas was used to maintain the desired pressure and anaerobic condition in the core-flooding apparatus throughout the experiment. The core-flood apparatus was maintained at the described experimental conditions for a period of 21 days. The cell counting was performed with the help of hemocytometer. Cells were stained using Trpan Blue and the total cells were counted using the following formula-

$$\text{Cells}/\text{ml}={\left(\text{Total cells counted}/\text{No of boxes counted}\right)}^{*}{\text{ Dilution factor}}^{*}10,000$$

### 2.10 Pathogenicity of microbial consortia

Since the study’s goal is to deploy lignite bioconversion into methane in the field, it is crucial to carry out the pathogenicity investigation to confirm that the consortiums being employed are suitable for industrial use. The pathogenicity test of THAA was studied by acute oral toxicity under EPA 712-C-96-322 OPPTS 885.3550 guidelines as described by Sharma et al. (2020). Forty mice—20 male and 20 female—were divided into the control and test dose groups in order to evaluate this. 8 mice (4 male and 4 female) were kept as control group. Mice were gavaged with the test substance once (1 mL per mouse). Three to 4 hours before and 2 hours after the test drug was administered, the mice were not given food. The experimental animals that were still alive at the end of the observation period were put to death. Gross necropsy was carried out, and each animal was closely inspected to look for bacterial culture. This study was performed by National Toxicology Centre (APT testing & Research PVT.LTD), Pune under EPA 712-C-96-322 OPPTS 885.3550 guidelines.

### 2.11 Data availability

All sequence data and metadata information are available through NCBI’s Sequence Read Archive under Bioproject PRJNA970089 (https://dataview.ncbi.nlm.nih.gov/object/PRJNA970089?reviewer=8bjo8u44povj5m32rhkvdu075b).

## 3 Results and discussion

### 3.1 Physico-chemical analysis of coal and formation fluid

The ultimate and proximate analysis data of the lignite samples from the well THAA and LJAY have been mentioned in Table 1. The biological conversion of lignite to methane is crucial for securing stable energy resources and sustainable development. Therefore, there have been several physicochemical and biological attempts to improve coal conversion to methane (Piao et al., 2018). Nevertheless, the methane yield depends upon the lignite composition. The data of ultimate and proximate analysis reveals its suitability for anaerobic digestion.

The physicochemical properties of the selected formation water samples (Tharad and Langnaj) were examined (Table 2). All samples showed pH in the range of 6.86–6.94. Total dissolved solids were estimated in all the formation water samples and were found to be in the range of 182–15788 mg/L.

Heavy metal analysis of formation water samples from both wells was done by the standard APHA method, wherein, except for iron, no other heavy metal was detected in the collected formation water samples. Detection of the heavy metals in the formation water is essential because, beyond the threshold levels of toxic ions, the growth of methanogens may be affected, thereby influencing the production of methane (Head et al., 2012). Toxic ions analysis of formation water samples was done by standard APHA 4500 and IS 3025 methods. In these methods, detection limits of the chloride were found at 9,659.41 mg/L in THAA and 3,208.93 mg/L in LJAY. Fluoride concentrations were found 5.59 mg/L in THAA and 0.95 mg/L in LJAY. Sulfate concentrations were found to be 183.21 mg/L in THAA and 175.89 mg/L in LJAY (Table 2). All these concentrations are in the range which doesn’t hinder the growth of methanogens. The physico-chemical analysis of formation water shows absence of heavy metal toxicity and ambient conditions for the growth of microbes (Rathi et al., 2019).

### 3.2 Enrichment studies

**FIGURE 1:**
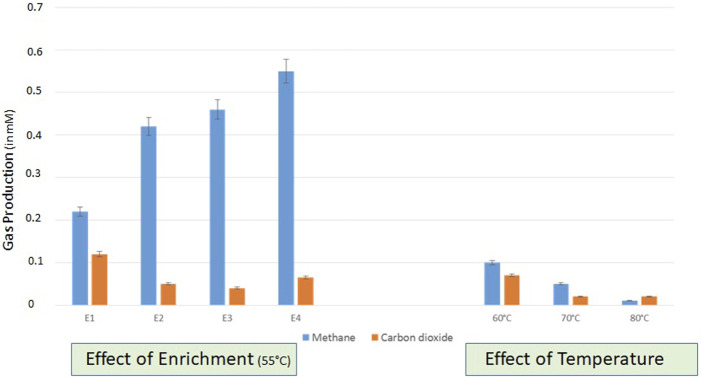
Assessment of gas production in different enrichments set uo at 55°C and right panel shows the effect of variable temperatures (60, 70, and 80°C) on the gas production (CH4 and CO2) by indigenous methanogenic consortia. Data was recorded after 20 days of incubation.

Enrichment studies were conducted with formation water collected from two lignite wells (THAA) to develop the indigenous bacterial consortia for bioconversion of lignite to methane. Due to the significant higher production of methane in the THAA well, the THAA samples were chosen for further assessment (Supplementary Figure S1). The amount of methane generated from the THAA formation water in each subsequent enrichment has been depicted in Figure 1. The first enrichment cycle comprised consideration of THAA cultures for onsite enrichment in two methanogen-specific media MPB containing 1%w/v of coal from the THAA well (lignite). It was in the fourth enrichment cycle where the maximum amount of methane was obtained.

The developed consortia from THAA formation water samples were further tested for a range of temperatures and salt concentrations to optimize the bioconversion of lignite to methane. The analysis was conducted at three temperatures (60°C, 70°C, and 80°C), and lignite samples from the THAA well were used with THAA inoculum. Figure 1 illustrates methane production at selected temperatures. From enrichment studies using Tharad inoculum, optimum methane bioconversion was observed at 55°C.

The analysis of the effect of variable concentrations of NaCl (0.5, 1.0, and 1.5 g/L) at selected temperatures (55°C, 60°C, and 70°C) showed the conditions of 55°C temperature and high NaCl concentration (1.5 gm/L) being the most suitable for methane production (Supplementary Figure S2). The optimized conditions supports the fesibility of the process on field for biogenic enhancement in methane production.

### 3.3 Bacterial community analysis

**FIGURE 2:**
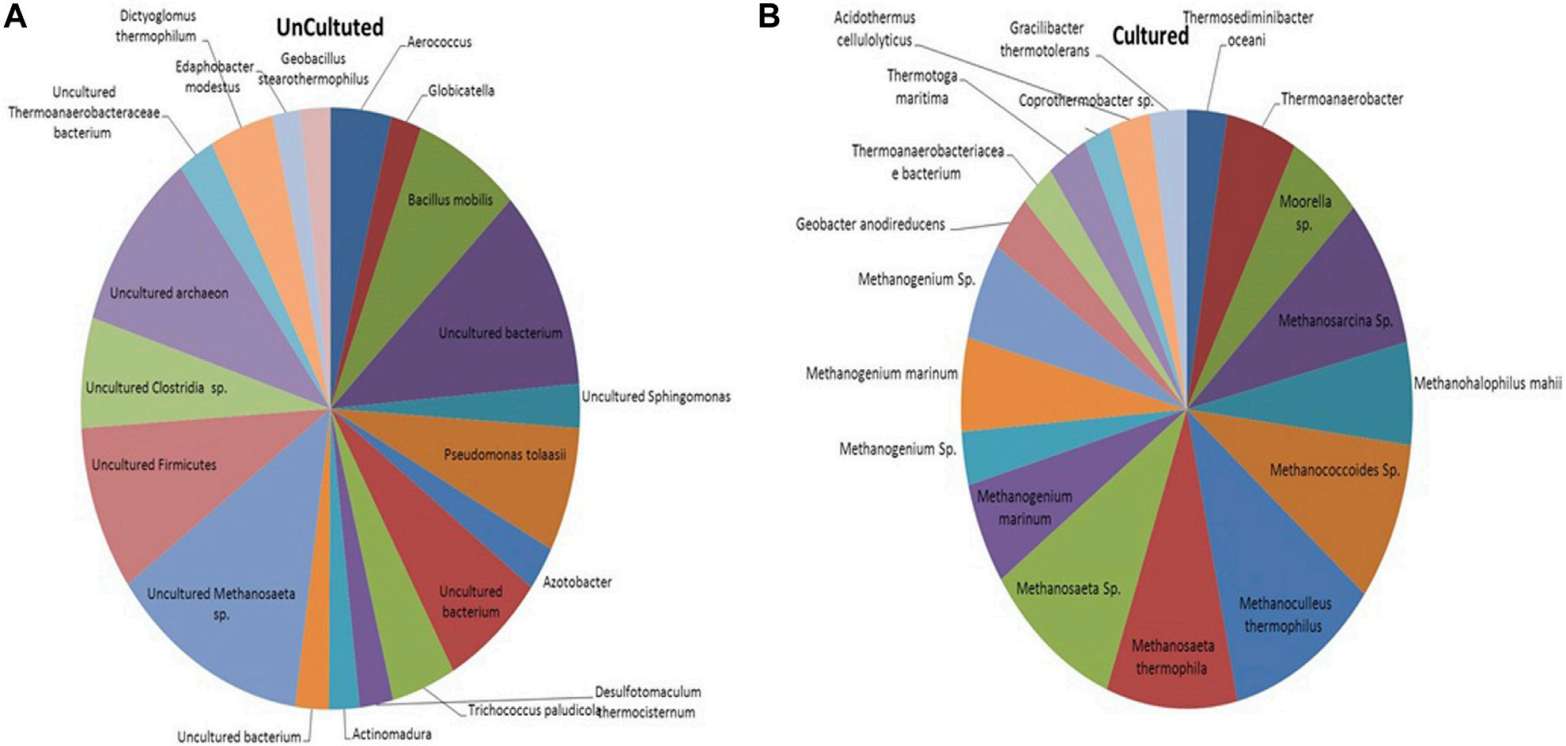
Figure illustrating the microbial community composition present in the **(A)** Formation water sample and **(B)** Enriched culture sample of THAA well, Tharad.

The microbial communities present in the formation water sample and enrichment culture of the THAA well (Tharad) have been illustrated in Figure 2. The figure shows the rich diversity of bacterial and archaea members in the formation water. The analysis shows the presence of various uncultured members of Methanosaeta, Firmicutes, Clostridia, Archaeon, and, Thermoanaerobacteraceae as the dominant community members in the formation water. Previous studies have reported the presence of Firmicutes, Clostridia, Archaeon, and, Methanosaeta in the formation water extracted from coal beds in various parts of the world. The finding suggests their key role in methane generation and the ability of microbes to survive in extreme environments (Gründger et al., 2015; Beckmann et al., 2019; Rathi et al., 2019). *Dictyoglomus thermophillum* is a chemoorganotroph and is extremely thermophilic. It plays an essential role in the degradation of organic matter, which is a crucial step for the generation of methane (Coil et al., 2014). Members of the class *Clostridia* are known to be involved in hydrogen-producing mechanisms (Gomez-Flores et al., 2017; Wang and Yin, 2021). Hydrogen acts as the limiting factor in the hydrogenotrophic methanogenesis pathway; therefore, the presence of these members may immensely impact the gas production in the wells (Kakuk et al., 2021). Members of class *Dictyoglomus* are abundantly present in producing wells. Members of *Dictyoglomus* are extremely thermophilic, chemoorganotrophic, and obligate anaerobes (Coil et al., 2014).

In the case of cultured adapted microbial consortia, the diversity was high of archaea groups. Dominant members belong to the order Methanosarcinales and Methanomicrobiales. Members like Methanogenium, Methanosaeta, Methanocullens, Methanococcoides, Methanohalophilus, Methanosarcina, and others. The dominance of methanogens in the enriched media shows its suitability for the process. The enriched culture shows presence of Acidothermus cellulyticus, a bacteria known for hydrolysis of organic matter (Shivlata and Tulasi, 2015). Members like Thermoanaerobacteriacea, Thermoanaerobacter, Thermotoga, and Thermosediminibacter are found in the enrichment samples. These members belong to the thermophilic group (Basen et al., 2018; Counts et al., 2017). The enrichment of methanogens and thermophillic microbes shows compatibility of the media to support the growth of microbes in higher temperatures. Various other studies have also supported that the enriched media can support only a subpopulation that dominates in the specific selective conditions (Bonnet et al., 2020).

### 3.4 Optimized nutrient recipe for enhanced biogenic methane generation

**FIGURE 3:**
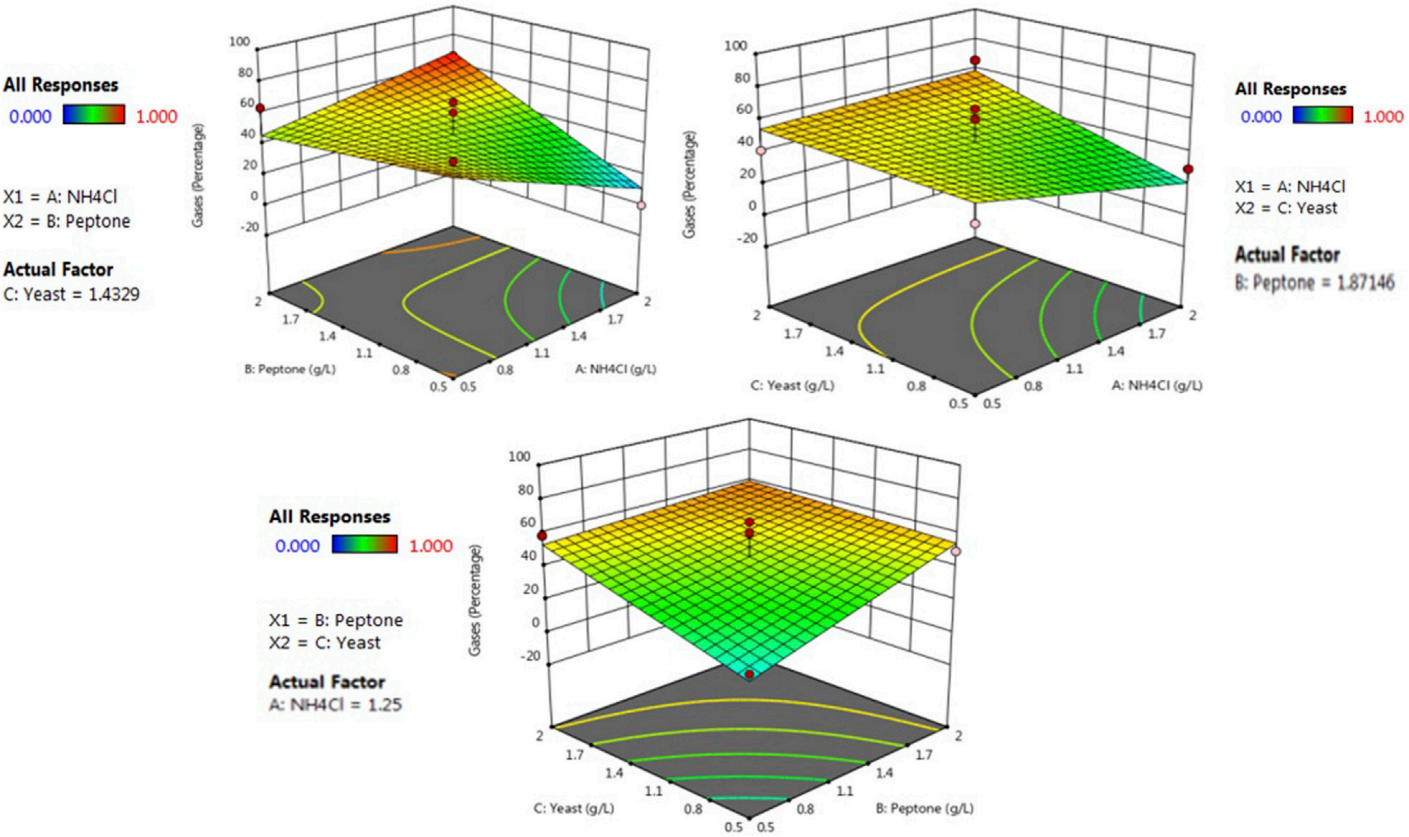
RSM contour plots of interactive effects of yeast extract, casein peptone, and ammonium chloride on methane production.

Three-dimensional response surface plots have been designed by keeping a variable at the center and the range of experiments differed with other variables. The effect of interaction for the production of methane gas and volatile fatty acid was analyzed from the three-dimensional surface curve plot. The variable interaction and the calculated response were plotted, and the three-dimension surface curves are shown in Figure 3; Supplementary Figure S3. The maximum response towards the methane gas production 0.897 mM (69.2%) was found to be with the concentration of yeast extract 1.43 g/L, peptone 1.87 g/L and ammonium chloride 1.25 gm/L. Similarly, the maximum response towards the volatile fatty acid production (27.12 mg/L) was found to be with the concentration of yeast extract 1.43 g/L, peptone 1.87 g/L, and ammonium chloride 1.25 g/L. Since the indigenous bacterial community involved in biological methane production are highly sensitive, it requires a specific concentration of each factor for its growth and production of methane, followed by volatile fatty acid. Similar findings with RSM were also shown by (Wu et al., 2017) in terms of a similar methane gas production trend found within the range. For maximal methane gas production, the optimum condition was found to be at 50°C temperature, the concentration of yeast extract 1.43 g/L, peptone 1.87 g/L and ammonium chloride 1.25 gm/L 7.2 pH, substrate concentration, 5 s agitation times.

### 3.5 Scale up study

**FIGURE 4:**
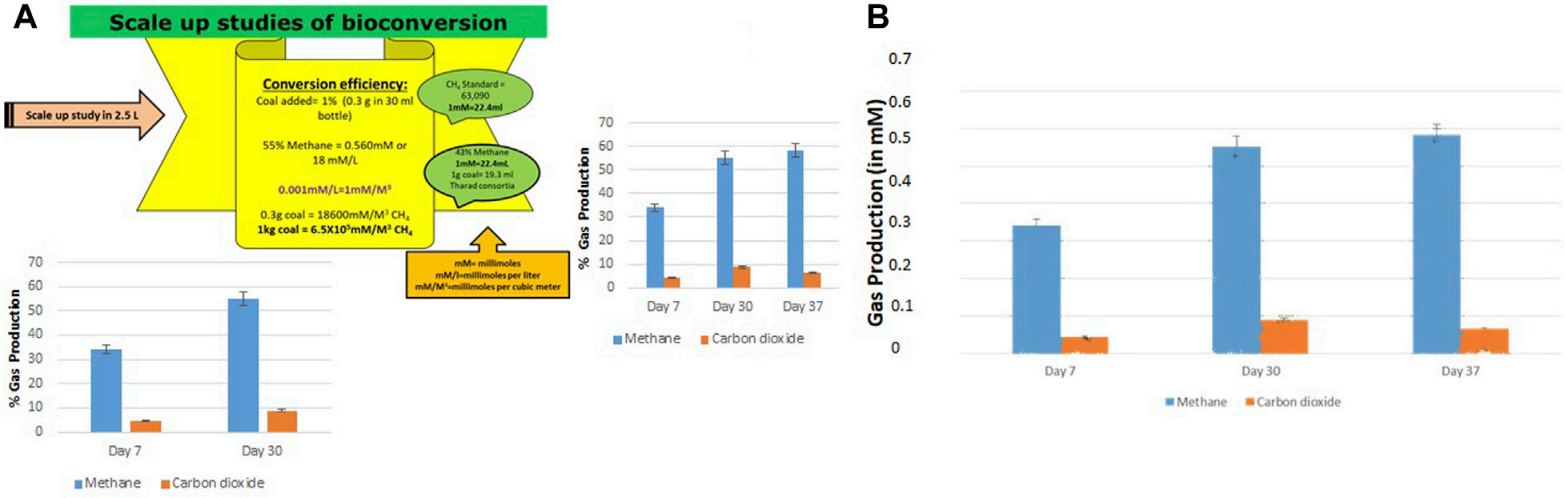
**(A)** Conversion efficiency calculation of scale up study and percentage production of methane and carbon dioxide during the scale-up (2.5 L) studies. **(B)** Gas production in scale up study in mM on Day 7, 30, and 37.

**FIGURE 5:**
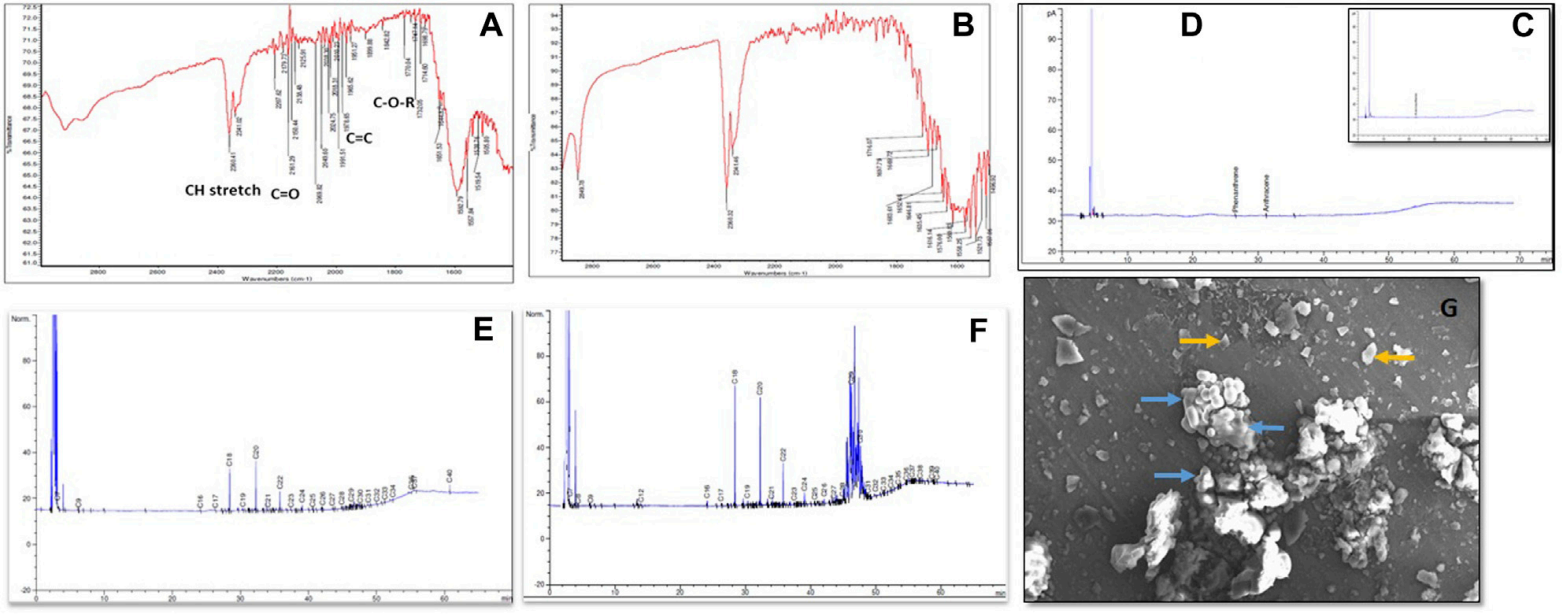
**(A)** FTIR spectra of residual Lignite (Before bioconversion process) **(B)** FTIR spectra of residual lignite (After the bioconversion process) C and D depicted the GC chromatograms representing aromatic hydrocarbon content in the coal where, **(C)** Before the bacterial treatment and **(D)** After the bacterial treatment. **(E)** Represents the aliphatic hydrocarbon in control, **(F)** Aliphatic hydrocarbons profile after bacterial treatment. **(G)** SEM depicted the interaction between lignite and bacteria at 920X magnification, where yellow arrows the coal particles, and blue arrows indicate microbial cells.

The bioconversion of lignite to methane was conducted at a large scale (2.5 L) with an optimized nutrient recipe for the THAA consortia and lignite as the sole carbon source. Figure 4 depicts the enhanced methane production by the Tharad consortia after incubation at regular intervals of 7–37 days. Figures 5A, B illustrate the lignite sample’s FTIR spectra before and after the bio consortia interaction. The recorded spectra showed that the peaks range between 2,360–2000 cm^−1^ are due to aliphatic groups in coal samples and from 1,600 to 1,655 cm^−1^ corresponds to aromatic C=C structure. The presence of C=O and C-O-R is revealed by the spectra obtained between the 1800–1,000 cm^−1^ region. In the treated condition, the aromatic C=C stretching indicates the presence of a high amount of carbon. The possibility is due to the reduction of oxygen through the transformation of C=O to CH_2_ or by decarboxylation of the matrix, which leads to an improved, calorific value. The results depicted that the bacterial treatment biologically converts the lignite into volatile compounds which can then be easily converted into methane.

Hydrocarbon degradation studies of the lignite sample have been represented in Figures 5C, D, which shows the alkane groups present in the lignite before and after the bacterial treatment. By observing the fragment pattern, a carbon chain from C_7_-C_40_ was detected. Major peaks were recorded for C_18_, C_20_, C_22_, C_29_, and C_30_. The results indicate partial depolymerization of geopolymers. A characterized THAA bacterial consortium was chosen for studying the interaction between lignite particles and the bacterial inoculum using scanning electron microscopy (SEM). Figure 5E reveals the morphology of bacteria as being coccus shaped, besides the interaction between the bacteria and lignite particles can also be seen.

### 3.6 Core flood

**FIGURE 6:**
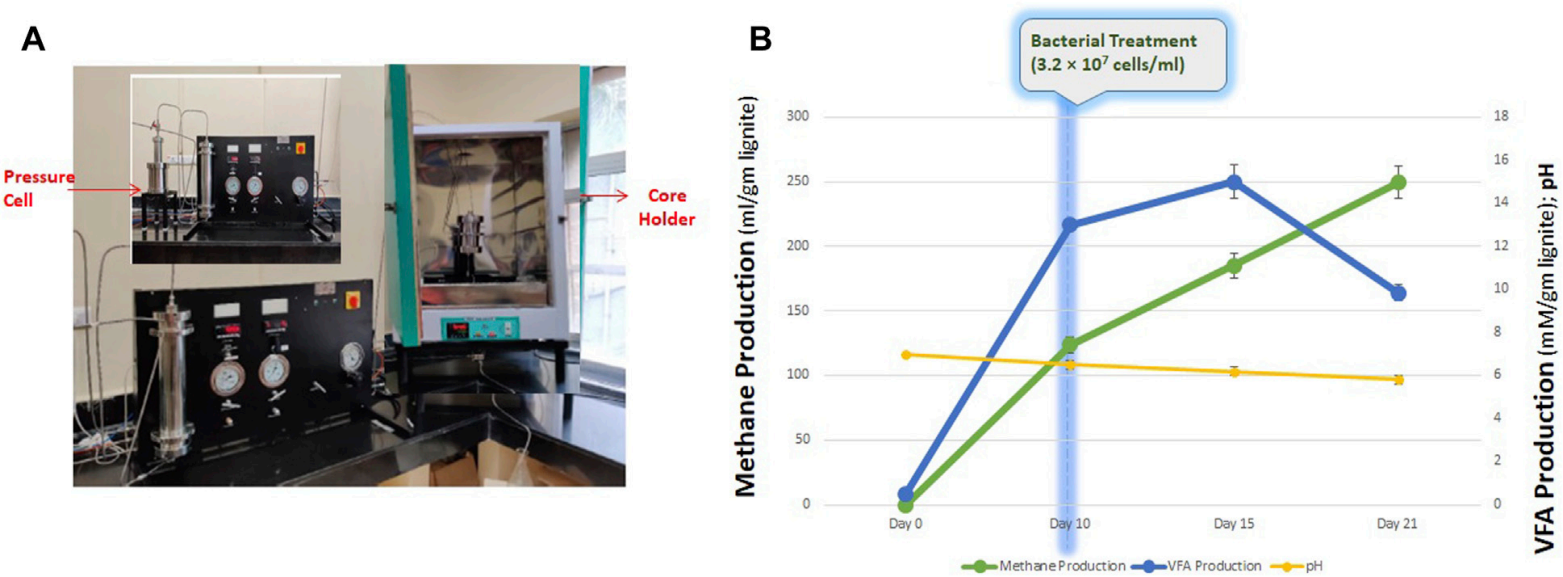
**(A)** Schematic diagram of core flood apparatus. **(B)** Production of methane, VFA and final pH of the core flood study.

After an incubation period of 21 days, the spent medium was collected in a sealed N_2_-flushed 1 L bottle. Under set experimental conditions, the consortium produced 249.51 ± 1.51 mL methane per gram of lignite. VFAs were also produced and detected in the collected spent medium sample. The total VFA content was 9.851 ± 0.367 mM, with Acetic Acid being the most abundant fatty acid. Acetic acid and propionic acids were 6.656 ± 0.169 mM and 1.170 ± 0.169 mM, respectively, along with fewer quantities of butyric acid and i-Valoric acid (Figure 6B). The pH of the spent medium collected was 5.82 ± 0.2, with the total cell count being 3.2 × $10^{7}$ cells/ml. The formation of various VFA in the process shows availability of substrate for the process of methanogenesis and its feasibility in the field. This shows the feasibility of lignite bioconversion to methane using THAA consortia when exposed to temperature and pressure conditions (Figure 6).

### 3.7 Pathogenicity of culture

Pathogenicity of microbial consortia obtained from formation water sample THAA, Tharad did not cause any mortality when administered in mice by oral route at the dose of 1.0 mL containing 1× $10^{8}$ CFU. Till the end of the study, all the Mice appeared normal and showed no clinical signs of intoxication after dosing. During the 21 days long observation period, no adverse effect was observed on the body weight gain of the treated mice. The necropsy and the study to find infectivity in tissues and organs of the mice made evident that THAA consortia did not induce any gross pathological alterations in any of the organs of the Mice and hence found to be safe.

## 4 Conclusion

Lignite is abundantly found in various parts of the world, but its low calorific value has less economic significance than other forms of coal. Therefore studies focusing on the effective bioconversion of lignite to methane carry great ecological importance. The present study showed around 0.897 mM production of methane gas through the bioconversion of lignite. The optimization study shows maximum methane production at the temperature of 50°C. The microbial diversity analysis showed diverse groups of bacteria and archaea present in the formation water. The enriched media showed their role in the effective bioconversion of lignite to methane. The analytical analysis shows the partial depolymerization of coal geopolymer. The SEM analysis showed the interaction of the microbes with the lignite surface.

After the optimization of conditions, the scale-up study revealed methane production as 2,800 mM/25 gm of coal, indicating the effective bioconversion of lignite to methane.

The results clearly show how this study is a step forward in the bioproduction of methane, as it utilizes the large lignite reserves, thus enhancing its ecological importance. In addition, the study was carried out successfully at high-temperature conditions, above 50°C, and at depth, too far down from the earth’s surface, compared to otherwise moderate conditions at the oil/coal wells situated in different regions of the world.

Besides, it uses indigenous nonpathogenic microbial consortia for bioconversion of lignite to methane, thus making it a viable choice for similar lignite-bearing oil wells across the country and ensuring there’s caused.

## Data Availability

The datasets presented in this study can be found in online repositories. The names of the repository/repositories and accession number(s) can be found here: https://www.ncbi.nlm.nih.gov/ the accession numbers are SRR24475124 and SRR24475123.
