# Supplementary material for: Laboratory investigation and core flood demonstration of enhanced biogenic methane generation from lignite
Source: Front Bioeng Biotechnol. 2024 Feb 19;12:1308308. doi: 10.3389/fbioe.2024.1308308 (PMC10910356; doi:10.3389/fbioe.2024.1308308)
Supplement: Supplementary file 1 [file DataSheet1.docx]

**Figure S1**: Represent the gas production data in 30th day of incubation where Langnaj coal showed 34% methane and Tharad showed 20% methane.


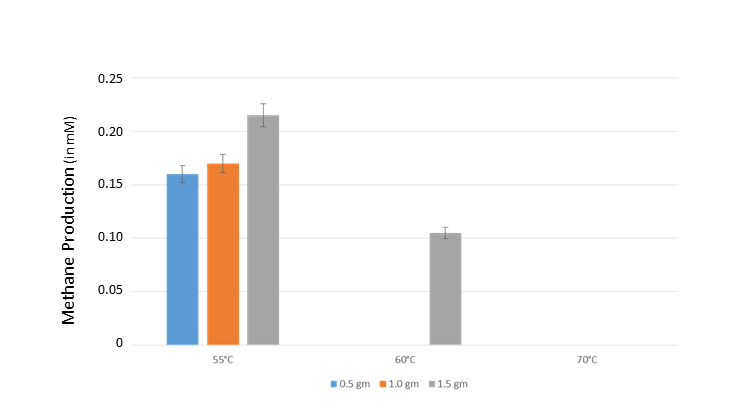


**Figure S2** Headspace methane production at variable temperature and salinity conditions by the microbial consortia developed from THAA formation water.


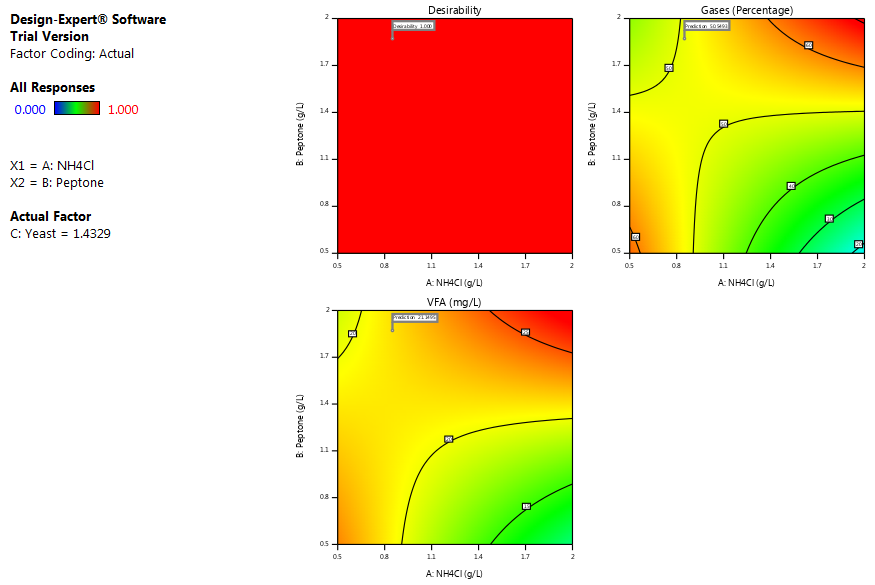

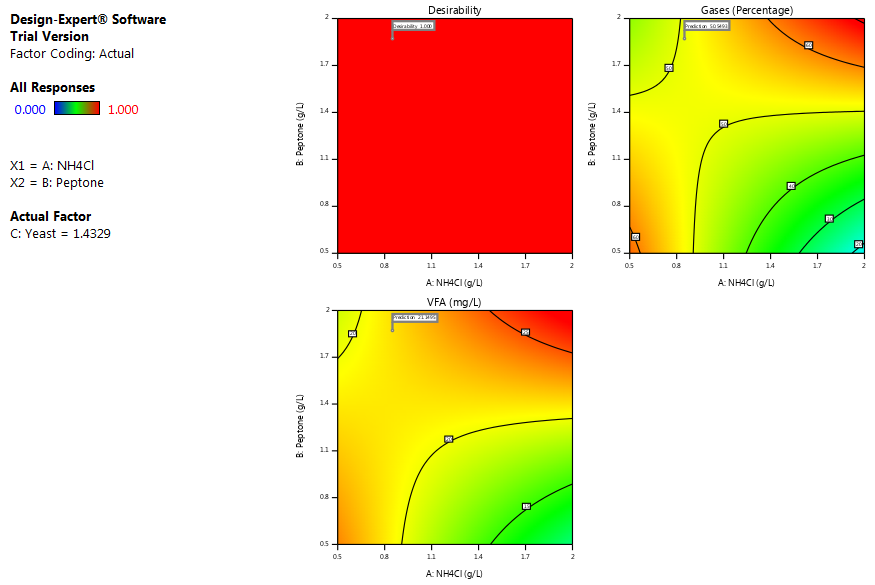

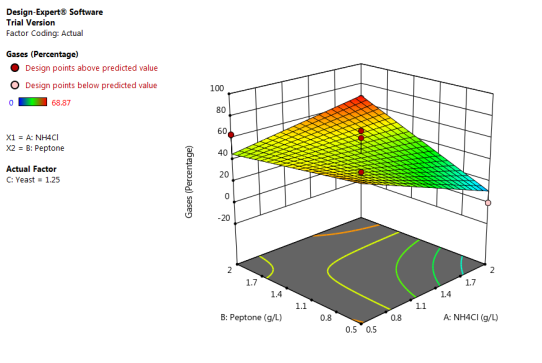

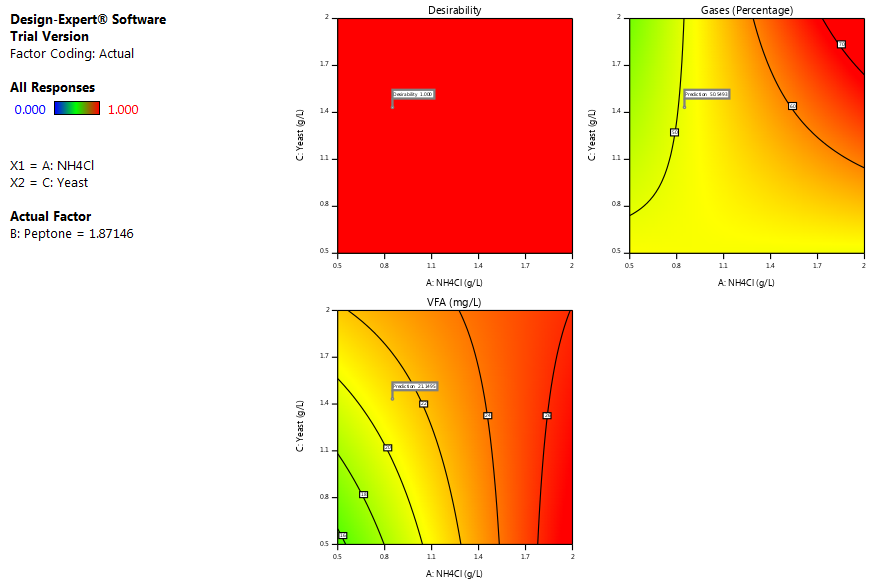

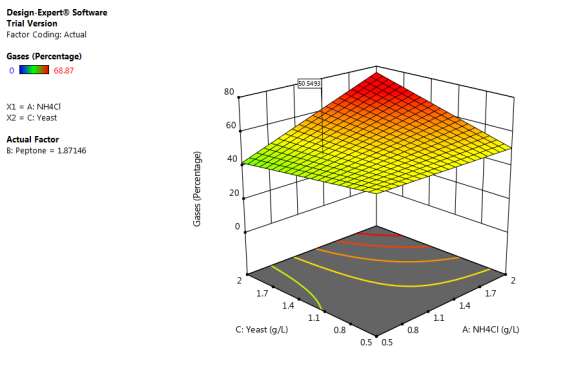

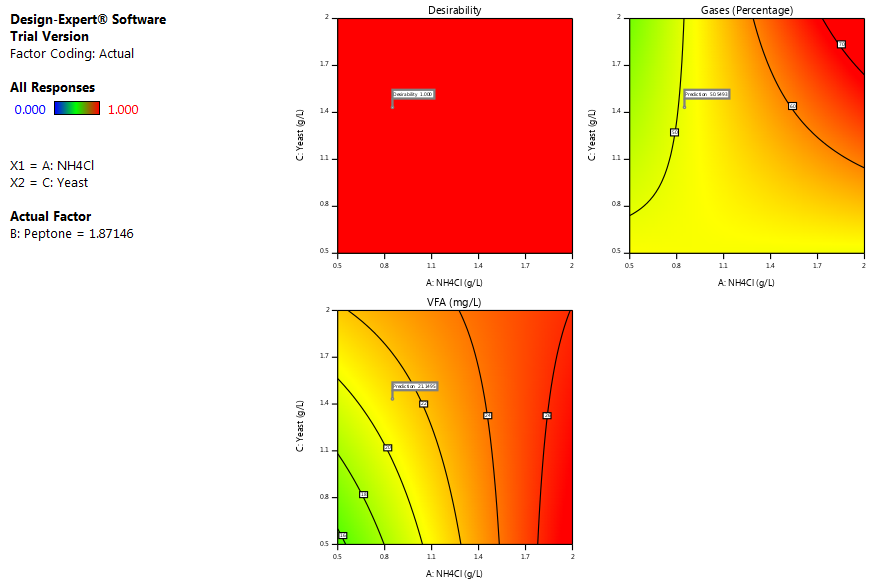

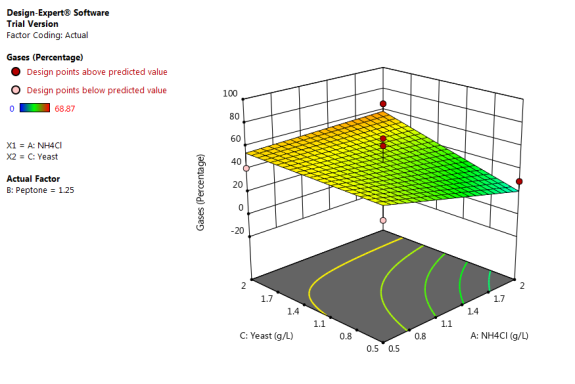


A B


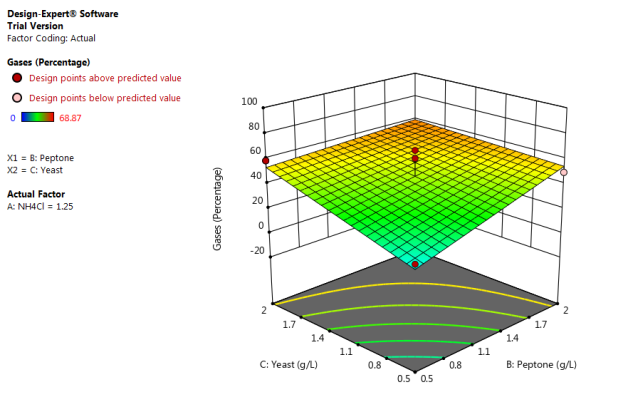

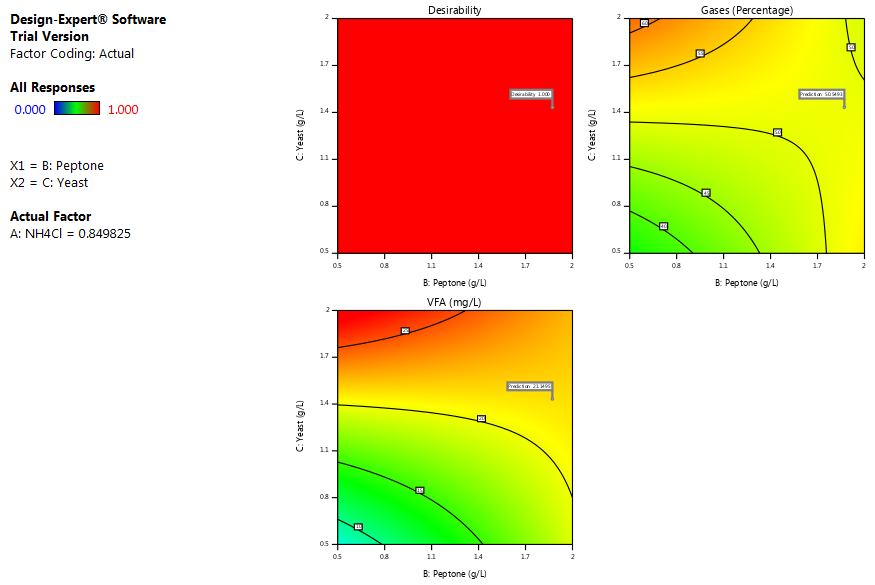

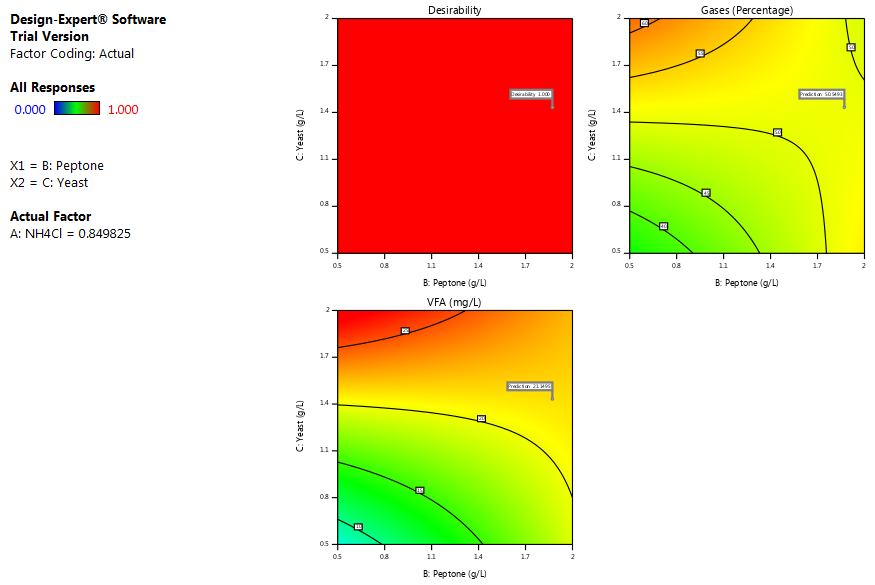

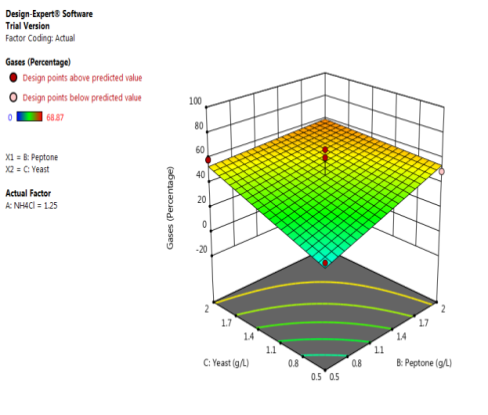


C

**Figure S3** RSM graph actual factor a) Yeast b) Peptone c) NH_4_Cl .

**Figure S4** Pathogenicity Report showing non-pathogenic nature of Consortia

Pathogenicity report showing non-pathogenic nature of Consortia
